# Cell-Free Supernatant Derived from a *Lactobacillus casei* BL23 Culture Modifies the Antiviral and Immunomodulatory Capacity of Mesenchymal Stromal Cells

**DOI:** 10.3390/biomedicines11061521

**Published:** 2023-05-24

**Authors:** Szabolcs Muzsai, Ore-Matan Maryanovsky, Roland Ander, Gábor Koncz, Anett Mázló, Attila Bácsi, Márta Tóth

**Affiliations:** 1 Department of Immunology, Faculty of Medicine, University of Debrecen, 4032 Debrecen, Hungary; muzsai.szabolcs@med.unideb.hu (S.M.); ormaryanov@gmail.com (O.-M.M.); anderroland54@gmail.com (R.A.); koncz.gabor@med.unideb.hu (G.K.); mazlo.anett@med.unideb.hu (A.M.);; 2 Gyula Petrányi Doctoral School of Clinical Immunology and Allergology, University of Debrecen, 4032 Debrecen, Hungary; 3 ELKH-DE Allergology Research Group, 4032 Debrecen, Hungary; 4 Doctoral School of Molecular Cell and Immune Biology, University of Debrecen, 4032 Debrecen, Hungary

**Keywords:** Lactobacillus, mesenchymal stromal cells, dendritic cells, immunomodulation, extracellular vesicles

## Abstract

Immune responses are highly complex and intricately regulated processes involving immune and non-immune cells in close direct and indirect contact with each other. These cells are highly sensitive to environmental signals, including factors derived from microbiota. Here, we demonstrate that the human microbiota member *Lactobacillus casei* (*L. casei*)-derived cell-free supernatant (CFS) enhances the sensitivity of mesenchymal-stromal-cell-like (MSCI) cells to viral stimuli and induces the development of dendritic cells (DCs) with anti-inflammatory and antiviral properties via pretreated MSCl cells. Our results showed that the production of INFβ and CXCL10 by MSCl cells upon viral stimulation was dependent on the presence of *L. casei*-derived extracellular vesicles in CFS during pretreatment. Moreover, *L. casei* CFS and/or poly (I:C)-conditioned MSCI cells altered the differentiation process of freshly isolated monocytes, as well as the developing DCs’ phenotype and functional activities, such as cytokine and chemokine secretion. Taken together, *L. casei* CFS contains factors which contribute to the pronounced antiviral response of MSCI cells, avoiding the development of inflammation via the induction of differentiation of anti-inflammatory DCs that retain their antiviral properties.

## 1. Introduction

Immune responses against viruses such as severe acute respiratory syndrome coronavirus 2 (SARS-CoV-2) can lead to severe inflammatory reactions which may have significant consequences on the patient’s further quality of life. Therefore, among the first-line candidates for the therapy of these diseases are the anti-inflammatory drugs, including non-steroidal ibuprofen or ketoprofen [1,2]. In addition to these, there are other options for reducing the inflammatory response. Manipulating the microbiota composition is a promising therapeutic approach for various diseases, including autoimmune disorders, metabolic diseases, inflammatory conditions, and even cancers.

There are a plethora of studies proving the anti-inflammatory/immunomodulatory effects of different Lactobacillus strains in various disorders, including inflammatory bowel diseases, such as Crohn’s disease and ulcerative colitis, and autoimmune diseases, such as rheumatoid arthritis and multiple sclerosis [3]. Lactobacilli and their metabolites also have beneficial effects on different viral infections, for example, by dampening the diarrhea symptoms caused by Rotaviruses or providing protection against respiratory infections [4,5]. The way in which the microbiota members contribute to the relief of the symptoms of certain diseases can be highly diverse; they can directly influence the function of immune cells that mediate inflammatory processes, and they can also contribute to the regulation of the immune response by conditioning other tissue-resident cells. It has also been shown that Lactobacilli [6,7] or Lactobacilli-derived conditioned media [8,9] can activate immune cells such as dendritic cells. Additionally, it has previously been documented that Lactobacilli can interact with mesenchymal stem/stromal cells (MSCs). Han et al. showed that *Lactobacillus* (*L.*) *reuteri* extracts could induce the proliferation and wound healing capacity of gingiva MSCs [10]. In another study, a *L. rhamnosus* culture supernatant in combination with bone marrow MSCs improved liver functions in an alcoholic steatohepatitis model in rats [11]. These observations emphasize the synergistic anti-inflammatory roles of microbiota components and MSCs in inflammatory conditions. MSCs are hypoimmunogenic cells by themselves maintaining the homeostasis of various tissues. The immunomodulatory actions of the MSCs are tightly regulated by the surrounding microenvironmental signals [12]. The licensing of the MSCs with different kinds of factors, such as pro-inflammatory cytokines, hypoxia, or pharmacological agents, is an effective therapeutic approach for various disorders, including inflammatory and degenerative diseases or graft rejection [13].

MSCs are multipotent, tissue-resident, spindle-shaped cells which can be isolated from multiple tissues, including bone marrow, adipose tissue, the umbilical cord and different vessels. Additionally, they can differentiate into many types of cells, such as adipocytes, chondrocytes, and osteoblasts [14]. Moreover, they have powerful direct and indirect immunomodulatory capacities. It has previously been reported that cell surface molecules including adhesion molecules, galectin-1, and programmed death ligand 1 (PD-L1) expressed by different types of MSCs can inhibit the activation of T cells [15,16,17]. Furthermore, MSCs produce numerous soluble mediators with paracrine and endocrine effects such as growth factors, cytokines, enzymes, and lipid mediators [12].

In addition to their immunosuppressive properties, MSCs can also sense pathogen- and danger-associated molecular patterns (PAMPs and DAMPs) and thus have antimicrobial activity against pathogens such as viruses. It has already been documented that MSCs have beneficial anti-inflammatory effects on viral infections such as COVID-19 [18,19]. Moreover, in cases of serious lung injury caused by influenza viruses, MSCs or MSC-derived metabolites reduced acute lung injury and weight loss and increased survival [20]. The antiviral response mediated by MSCs and their immuno-tolerogenic properties may synergistically affect the causes and symptoms of severe viral infections.

MSCs can produce cytokines and chemokines essential for viral clearance and, in addition to their direct effect, modulate the functions of other host cells involved in antiviral immunity [21]. Immunomodulation by MSCs affects almost all the immune cell types in the body, including adaptive T and B cells and innate immune cells such as granulocytes, NK cells, and monocytes [22]. MSCs may also influence the antiviral capacity of NK cells and cytotoxic T cells through the regulation of monocytes and DCs [21]. Monocytes are highly plastic cells; their differentiation and functions are tightly regulated by the local microenvironment. Since monocytes are not fully differentiated cells, their modulation is a favorable target for MSCs. It is known that the MSC cell line indirectly forces the differentiation of monocytes into CD1a^−^DC-SIGN^+^CD163^low^CTLA-4^+^ semi-matured moDCs, whereas these cells directly promote the differentiation of CD1a^−^DC-SIGN^low^CD163^high^ M2 macrophage-like cells in a steady state [23]. Additionally, it is widely accepted that MSCs generally silence the inflammatory features of moDCs upon viral or bacterial infection [24].

In this study, we aimed to investigate the effects of complete and extracellular-vesicle (EV)-depleted cell-free supernatant (CFS) derived from the probiotic *L. casei* BL23 on the ability of human MSC-like (MSCl) cells to respond viral stimuli. We also examined whether *L. casei* CFS- and/or poly (I:C)-conditioned MSCI cells alter the differentiation, phenotype, and function of human monocyte-derived DCs (moDCs).

## 2. Materials and Methods

### 2.1. Preparation of Cell-Free Supernatants (CFS) from Lactobacillus Strains

The human gut symbionts *L. casei* BL23 and *L. reuteri* ATCC PTA 6475 were cultured for 20 h in Difco™ de Man, Rogosa and Sharpe (MRS) liquid broth medium (BD Difco, Fisher Scientific, Co., LLC, Pittsburgh, PA, USA) at 37 °C without shaking. The bacterial cultures were then centrifuged at 14,000× *g* and adjusted to pH 7 with 2N NaOH, and the supernatants were sterile-filtered using a 0.2 µm filter. After that, the supernatants were aliquoted and stored at −70 °C until further use. EVs were removed from a portion of the *L. casei* supernatants via ultracentrifugation at 100,000× *g* for 2.5 h at 4 °C.

### 2.2. Generation and Maintenance of Human moDC Cultures

Peripheral blood mononuclear cells were obtained from blood samples using Ficoll-Paque Plus (Amersham Biosciences, Uppsala, Sweden) gradient centrifugation. To purify the monocytes from the PMBCs, positive magnetic cell separation was performed using anti-CD14-conjugated microbeads (Miltenyi Biotec, Bergisch Gladbach, Germany) according to the manufacturer’s instructions. The isolated monocytes were plated at 1 × 10^6^ cells/mL in RPMI-1640 medium (Merck KGaA, Darmstadt, Germany) supplemented with 10% FCS, 1% Penicillin-Streptomycin, and 1 g/L L-glutamine (all from Gibco, Thermo Fisher Scientific, Waltham, MA, USA). For the induction of differentiation, 100 ng/mL IL-4 (PeproTech EC, London, UK) and 80 ng/mL GM-CSF (Gentaur Molecular Products, Brussels, Belgium) were added to the cells.

### 2.3. Maintenance of the Mesenchymal Stromal Cell-like Cell Line

In our experiments, we used a mesenchymal stromal cell-like (MSCl) cell line provided by the work group of Prof. Dr. Balázs Sarkadi and Dr. Ágota Apáti (Semmelweis University, Budapest), the collaboration partners of the Department of Immunology (University of Debrecen, Debrecen). The human embryonic stem cell lines (HUES9), which form the basis of this cell line, were provided to the work group of Prof. Dr. Balázs Sarkadi by Dr. Douglas Melton of the Howard Hughes Medical Institute. The MSCl cells were used according to ethical permission (6681/2012/EHR). The cell line was obtained in liquid nitrogen in a frozen state. After thawing, the MSCl cells were washed twice with DMEM medium (Thermo Fisher Scientific) containing 10% FCS, 1% Penicillin-Streptomycin, and 1 g/l L-glutamine (All from Thermo Fisher) and then cultured in the same medium at 37 °C with 5% CO_2_ in a humidified air incubator.

### 2.4. Experimental Models

**Figure 1:**
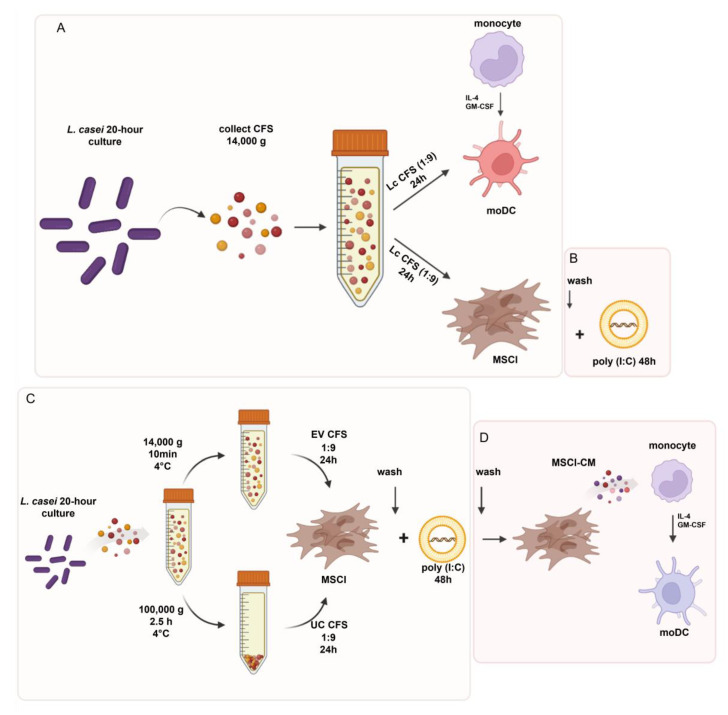
Experimental setups used in the study. Cell-free supernatant (CFS) of *L. casei* subjected to 20 h of culture was collected at 14,000× *g* and used in RPMI-1640 medium at the ratio of 1:9 to precondition monocyte-derived DCs and MSCl cells for 24 h (**A**). In the further experiments, MSCl cells were preconditioned with *L. casei* CFS and then washed, and the cells were activated with 1 µg/mL poly (I:C) in complex with LyoVec for 48 h (**B**). A portion of *L. casei* CFS was ultracentrifuged at 100,000× *g* for 2.5 h at 4 °C to deplete the extracellular vesicles (EVs) from the bacterial CFS. This ultracentrifuged (UC), EV-depleted CFS, in parallel with the EV-containing bacterial supernatant, was used in RPMI-1640 medium at the ratio of 1:9 to precondition the MSCl cells for 24 h, followed by washing, and the cells were activated with 1 µg/mL poly (I:C) in complex with LyoVec for 48 h (**C**). UC and EV-containing CFSs of *L. casei* were used in RPMI medium at the ratio of 1:9 to precondition MSCl cells for 24 h and then washed, and the cells were activated with 1 µg/mL poly (I:C) in complex with LyoVec for 48 h. The pretreated and activated cells were washed and cultured in RPMI medium for another 48 h. The obtained MSCl-conditioned medium (MSCl-CM) from viral-ligand-activated MSCl cells were used for the differentiation of moDCs for 3 days (**D**). Figure was created with the online version of BioRender.

Next, 5-day monocyte-derived dendritic cells (moDCs) and MSCl cells were exposed to *L. casei* cell-free supernatant (CFS) in RPMI medium at a ratio of 1:9 for 24 h (Figure 1A). Based on our initial results obtained with the moDCs and MSCl cells, the further experiments were carried out with the MSCl cells alone.

After 24 h, the bacterial CFS was washed from the MSCl cultures. Aiming to mimic rotavirus infection, 1 μg/mL poly (I:C) (InvivoGen, San Diego, CA, USA) was used in complex with LyoVec (InvivoGen) to activate the MSCl cells for 48 h (Figure 1B). These steps were repeated using the EV-containing (EV CFS) and EV-depleted (UC CFS) *L. casei* CFSs (Figure 1C). The cells were used in analyses, or the viral stimulus was washed from the MSCl cells, and the cells were rested in RPMI medium for an additional 48 h to obtain conditioned media using pretreated and activated stromal cells. These MSCl-conditioned media (MSCl-CM) were used to study the indirect effects of the preconditioned and preactivated MSCl cells on moDC differentiation (Figure 1D). Hence, to examine the effects of bacterial CFS-exposed MSCl cells, RPMI medium was supplemented with MSCl-CM at a ratio of 1:3, and the monocytes were cultured in the presence of IL-4 and GM-CSF for 3 days (Figure 1D).

### 2.5. Flow Cytometry

To study the phenotypic appearance of moDCs and MSCl cells exposed to the bacterial supernatant, flow cytometry was performed using CD83-fluorescein isothiocyanate (FITC), CD86-phycoerythrin (PE), CD80-PE, and HLA-DQ-FITC antibodies for the moDCs, and CD90-FITC, CD73-PE, CD44-peridinin chlorophyll protein (PerCP), CD49b-allophycocyanin (APC), CD105-FITC, CD29-PE, CD54-FITC, and CD49a-PE antibodies for the MSCl cells (all from BioLegend, San Diego, CA, USA).

Phenotypical analysis of the moDCs treated with MSCl-CM was accomplished using CD14-FITC, CD209/DC-SIGN-PE, CD86-PE, CD163-PE, and HLA-DQ-FITC antibodies (all from BioLegend).

It has previously been demonstrated that T cells can be indirectly activated by DCs [25]. To examine the indirect link between DCs and T cells, allogenic monocyte-depleted peripheral blood lymphocytes (PBLs) were exposed to supernatants from the MSCl-CM-treated moDCs at a ratio of 1:1 and then activated with 20 ng/mL phorbol 12-myristate 13-acetate (PMA) and 1µg/mL Ionomycin (both from Merck KGaA). The phenotypic appearance of T cells was measured using CD3-FITC, CD4-PE, CD8-PE (BioLegend), and IFNγ-APC (Sony Biotechnology Inc., San Jose, CA, USA) antibodies.

An ACEA NovoCyte 2000R cytometer (Agilent, Santa Clara, CA, USA) was used to detect the fluorescence intensities of the stained cells and unstained controls. The obtained data were analyzed using FlowJo software version X.0.7 (Tree Star Inc., Ashland, OR, USA).

### 2.6. Measurement of the Cytokine Concentrations via Enzyme-Linked Immunosorbent Assay (ELISA)

Supernatants of the MSCl cells and moDCs were collected, and the production of cytokines and chemokines was determined via an enzyme-linked immunosorbent assay using CXCL10, IL-1β, IL-6, IL-8, IL-12, TGF-β, and TNFα OptEIA kits (Beckton Dickinson, BD Biosciences, Franklin Lakes, NJ, USA) or a human IFNβ kit (R&D Systems, Minneapolis, MN, USA) according to the manufacturers’ instructions.

IL-8, IL-6, IL-1β, TNFα, IFNβ, and CXCL10 production was measured in the MSCl-supernatants. Furthermore, the concentrations of CXCL10, IL-1β, IL-6, IL-8, IL-10, IL-12, TGF-β, and TNFα cytokines (Beckton Dickinson, BD Biosciences) were detected in the moDC-supernatants.

Absorbance was detected with a Synergy™ HT Multi-Detection Microplate Reader (Bio-Tek Instruments, Winooski, VT, USA) at 450 nm using KC4 v.3.4. software. The data were analyzed with Microsoft Excel.

### 2.7. RNA Isolation, DNase Treatment, Reverse Transcription, and Real-Time Quantitative Polymerase Chain Reaction (PCR)

Briefly, total RNA from MSCl cells treated with *L. casei* and *L. reuteri* CFSs and activated with poly (I:C) was isolated using guanidine isothiocyanate solution (TRIzol, Thermo Fisher). To degrade the genomic DNA residues, the RNA samples were subjected to DNase treatment using DNase I enzyme (Ambion, Thermo Fisher). Isolated and DNA-free RNA was then reverse-transcribed into cDNA using a High-Capacity cDNA Reverse Transcription Kit (Applied Biosystems, Thermo Fisher).

Quantitative PCR was performed using gene-specific TaqMan assays (Thermo Fisher) at a final volume of 12.5 μL in duplicate using Dream Taq DNA polymerase (Thermo Fisher) and ABI StepOnePlus real-time PCR instruments (Applied Biosystems, Thermo Fisher). The expression of the examined genes was normalized to the h36b4 housekeeping gene utilizing specific primers and a probe (Integrated DNA Technologies, Coralville, IA, USA). The cycle threshold values were determined using the StepOne Software v2.1 (Applied Biosystems, Thermo Fisher) and Excel programs.

### 2.8. Statistical Analysis

A comparison between the two groups was carried out using the unpaired, two-tailed Student’s *t*-test, applying GraphPad Prism 6 software (GraphPad Software Inc., San Diego, CA, USA). Comparisons of two independent variables were performed via two-way analysis of variance (ANOVA) followed by Tukey’s post hoc test using GraphPad Prism 6 software. The results are expressed as the mean ± SD. The values * *p* < 0.05, ** *p* < 0.01, *** *p* < 0.001, and **** *p* < 0.0001, compared to control cells not activated with poly (I:C), and # *p* < 0.05, ## *p* < 0.01, ### *p* < 0.001, and #### *p* < 0.0001, compared to control cells not treated with CFS, were considered statistically significant. The results of the statistical comparison between the EV-containing and EV-depleted samples are illustrated in figures with the precise *p*-values. Our results are based on at least three independent measurements. 

## 3. Results

### 3.1. L. casei CFS Induces Inflammatory Cytokine Production by moDCs while Triggering IL-6 and IL-8 Production by MSCl Cells

Compounds/metabolites derived from microbiota members interact with immune and non-immune cells and influence their responses to pathogens such as viruses. However, the exact direct and indirect mechanisms exerted by particular microbiota members, such as different Lactobacillus strains, have not been fully uncovered. Therefore, we aimed to explore the effects of *L. casei* CFS on DCs, an immunogenic cell type, and on MSCs, a proved hypoimmunogenic cell type.

**Figure 2:**
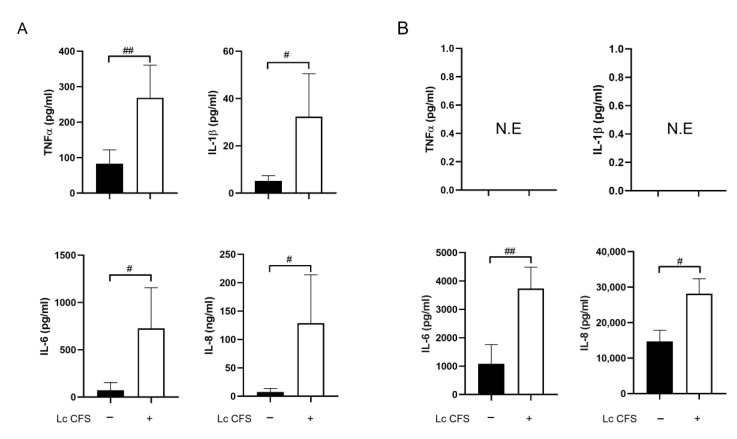
*L. casei* CFS modulates moDCs’ and the MSCl cells’ cytokine and chemokine secretion. MSC-like cells and 5-day moDCs were treated with CFS from *Lactobacillus casei* for 24 h, and then the production of TNFα, IL-1β, IL-6, and IL-8 by moDCs (**A**) and MSCl cells (**B**) was measured using the ELISA method. Results from 3–5 independent measurements are presented as means ± SD. N.E stands for not expressed. Student’s paired *t*-test was used for statistical analysis. Significance was defined as # *p* < 0.05 and ## *p* < 0.01 in comparison to control cells not treated with CFS.

First, we aimed to compare the DC- and MSC-activating capacities of *L. casei* CFS. To achieve this, 5-day moDCs and MSCl cells were incubated with *L. casei* CFS (1/9) for 24 h (Figure 1A). The concentrations of inflammatory cytokines, TNF-α, IL-1β, and IL-6, and the chemokine IL-8 in the harvested supernatants of the moDCs and MSCl cells were measured. We found that the *L. casei* CFS induced significantly higher inflammatory cytokine and chemokine secretion by the moDCs (Figure 2A). Moreover, the *L. casei* CFS significantly upregulated the expression of the activation marker CD83 and co-stimulatory CD86 cell surface molecules by moDCs (Figure S1A). The expression of the co-stimulatory CD80 and the HLA-DQ molecule was also increased but in a non-significant manner (Figure S1A). 

Despite the fact that MSCs from different tissues are able to express TNF-α [26], MSCl cells treated with *L. casei* CFS could not produce TNF-α and IL-1β, as these cytokines are instead priming for MSCs, eliciting their robust immunomodulatory potential [27]. Importantly, *L. casei* CFS treatment induced a significant elevation in the IL-6 and IL-8 concentrations in MSCl cells (Figure 2B). Since, in our experimental system, *L. casei* CFS did not cause inflammatory TNFα and IL-1β cytokine production by the MSCl cells but prompted moDC-activation, we examined the effects of *L. casei* CFS on the MSCl cells’ antiviral and immunomodulatory responses in further experiments.

To exclude the possibility that the MSCl cells did not differentiate in any direction in the presence of *L. casei* CFS, we checked the phenotypic characteristics of the cells. Importantly, we did not find any alterations in the expression of the typical MSC markers, CD90, CD73, CD44, CD105, CD29, CD49b, CD54, and CD49a, in the CFS-treated MSCl cells (Figure S1B).

Collectively, our results indicate that *L. casei* CFS possesses moDC-activating potential; however, it does not induce inflammatory TNF-α and IL-1β secretion or phenotypical changes in MSCl cells.

### 3.2. L. casei CFS Enhances the Sensitivity of MSCl Cells to Viral Stimulus and Induces the Increased mRNA Expression of Antiviral IFNβ Cytokine

**Figure 3:**
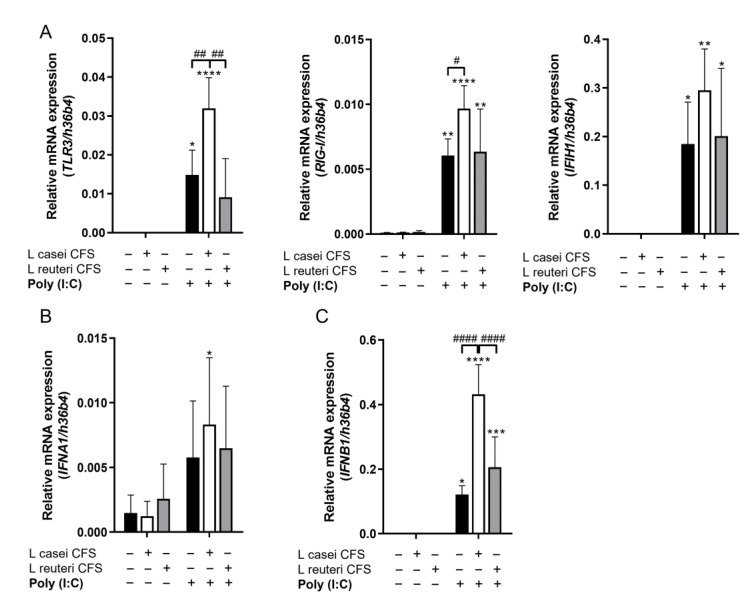
*L. casei* CFS increases the expression of virus-recognizing receptors and type I interferon genes by MSCl cells, whereas *L. reuteri* CFS does not. MSCl cells were treated with CFSs from *L. casei* or *L. reuteri* for 24 h and then washed and activated with 1 µg/mL poly (I:C) for 48 h. The mRNA expression levels of the *TLR3*, *RIGI*, and *IFIH1/MDA5* pattern recognition receptors (**A**), as well as *IFNA1* (**B**) and *IFNB1* (**C**), were measured via RT-qPCR. Figures show the means of relative expression of the target genes in reference to the *h36b4* household gene. Results from 4 independent measurements are presented as means ± SD. Two-way ANOVA with Tukey’s multiple comparison test was used for statistical analysis. Statistical significance was expressed as * *p* < 0.05, ** *p* < 0.01, *** *p* < 0.001, and **** *p* < 0.0001 compared to control cells not activated with poly (I:C), as well as # *p* < 0.05, ## *p* < 0.01, and #### *p* < 0.0001 for the comparison of CFS-treated and non-treated cells.

In the next step, we aimed to compare the effects of *L. casei* and *L. reuteri* CFSs on the MSCl cells’ antiviral response. Hence, MSCl cells were preconditioned with the *L. casei* and *L. reuteri* CFSs for 24 h. Then, the CFSs were removed through washing, and a synthetic viral ligand, poly (I:C) in complex with LyoVec transfection reagent, was added to the MSCl cells for 48 h (Figure 1B). After the incubation with the synthetic ligand, we first investigated the intracellular PRRs, namely, *TLR3*, *RIGI*, and *IFIH1/MDA5* expression, at the mRNA level in the MSCl cells in the presence and absence of the bacterial CFSs. The CFSs themselves did not induce any changes in *TLR3*, *RIGI*, or *IFIH1/MDA5* expression, while the poly (I:C) treatment induced significantly increased expression of these receptors (Figure 3A). Furthermore, preconditioning with *L. casei* CFS resulted in significantly higher *TLR3* and *RIGI* expression in the poly (I:C)-activated MSCl cells compared to viral stimulus alone. *IFIH1/MDA5* expression also showed a non-significant elevation in the MSCl cells upon co-activation with *L. casei* CFS and poly (I:C). In contrast, *L. reuteri* CFS could not induce any changes in the expression of the viral-sensing receptors, as compared to the poly (I:C)-activated control cells (Figure 3A). Based on our findings, the preconditioning of MSCls with Lc CFS modulates PRR expression in a synergistic manner.

Since *L. casei* CFS modified PRR expression in the MSCl cells, we studied the effects of the CFSs on antiviral cytokine expression by MSCl cells. MSCl cells exposed to the lactobacterial CFSs alone expressed low levels of *IFNA1* (Figure 3B) and did not express *IFNB1* (Figure 3C). In contrast, the poly (I:C) stimulus elevated the expression of both antiviral cytokines, though in a non-significant manner in the case of *IFNA1*. The poly (I:C)-stimulus-induced expression of *IFNA1* was not altered by the *L. casei* or *L. reuteri* CFS (Figure 3B). On the other hand, *L. casei* CFS could further elevate the expression of *IFNB1* in the poly (I:C)-stimulated MSCl, cells whereas *L. reuteri* could not (Figure 3C).

Our results suggest that *L. casei* CFS sensitizes MSCl cells to the poly (I:C) stimulus and prompts the expression of antiviral *IFNB1* in MSCl cells.

### 3.3. L. casei-Derived EVs Alter IFNβ and CXCL10 Production by MSCl Cells

**Figure 4:**
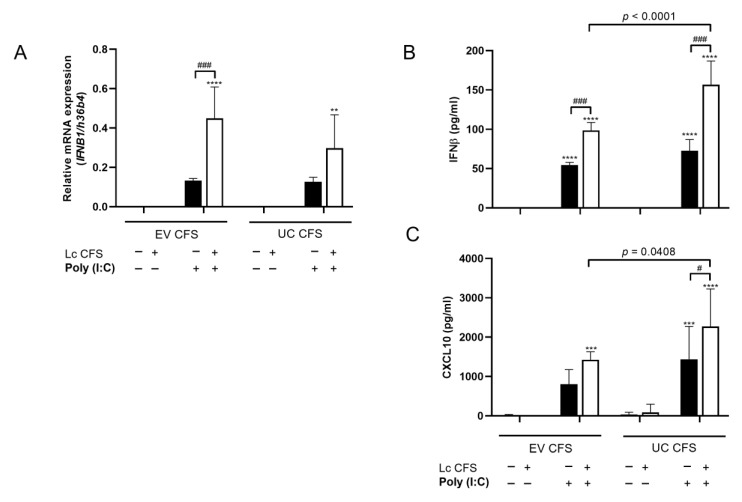
The gene expression of IFNβ does not change but IFNβ and CXCL10 secretion is increased by MSCl cells after the removal of extracellular vesicles from the bacterial CFS. MSCl cells were exposed to EV-containing and UC CFSs of *L. casei* for 24 h and then washed and activated with 1 µg/mL poly (I:C). The gene expression level of IFNβ (**A**) was determined via qRT-PCR. Figures show the mean of relative expression of the IFNβ gene in reference to the h36b4 household gene. Results are based on 3 independent measurements ± SD. The secretion of IFNβ (**B**) and CXCL10 (**C**) by pretreated and activated MSCl cells was measured via ELISA. Figures show the mean of concentrations from 4 independent experiments ± SD. Two-way ANOVA with Tukey’s multiple comparison test was used for statistical analysis. Significance was defined as ** *p* < 0.01, *** *p* < 0.001, and **** *p* < 0.0001 for the poly (I:C)-treated and untreated cells and # *p* < 0.05 and ### *p* < 0.001 for the comparison of CFS-treated and untreated cells. A significant difference in INFβ secretion between the EV-containing and EV-free CFS-treated MSCl cells upon poly (I:C) activation was defined as *p* < 0.0001.

It was previously shown that EVs derived from vaginal Lactobacilli suppress HIV-1 infection [28]. To study whether vesicles derived from *L. casei* CFS may affect the MSCl cells’ antiviral response, EVs were depleted from the bacterial supernatant via ultracentrifugation. After that, the MSCl cells were preconditioned with the EV-containing and EV-depleted ultracentrifuged (UC) *L. casei* CFSs for 24 h. After washing, the cells were activated with a poly (I:C)–LyoVec complex for 48 h (Figure 1C). First, we examined the mRNA expression of *IFNB1* by MSCl cells pretreated with EV-containing and UC *L. casei* CFSs. Both CFSs induced significantly elevated *IFNB1* expression by the MSCl cells upon poly (I:C) exposure as compared to the CFS alone (Figure 4A). The EV-containing *L. casei* CFS caused a significant increase in *IFNB1* mRNA expression by the poly (I:C)-exposed MSCl cells as compared to the viral stimulus alone (Figure 4A). However, pretreatment with the UC *L. casei* CFS and subsequent activation with poly (I:C) did not induce elevated *IFNB1* expression by the MSCl cells in comparison with the poly (I:C)-exposed MSCl cells (Figure 4A).

Next, we measured IFNβ production by the EV-containing and UC *L. casei*-CFS-exposed MSCl cells upon poly (I:C) activation. The MSCl cells released IFNβ upon poly (I:C) stimulation, and preconditioning with both *L. casei* CFSs further increased the production of this cytokine as compared to the viral stimulus alone (Figure 4B). Surprisingly, the removal of EVs from the *L. casei* CFS significantly enhanced the IFNβ production compared to its EV-containing counterpart after viral ligand administration (Figure 4B). The secretion of CXCL10—a crucial chemokine in viral infections—is induced by many types of interferons, including IFNβ [29]. Therefore, we measured CXCL10 production by the EV-containing and UC *L. casei*-CFS-exposed MSCl cells upon poly (I:C) stimulation. We found that the secretion of CXCL10 was regulated in a manner similar to IFNβ, i.e., the UC *L. casei* CFS induced elevated CXCL10 production by the poly (I:C)-activated MSCl cells as compared to the EV-containing CFS (Figure 4C).

These results indicate that the removal of EVs from the bacterial CFS influenced the modulatory effects of the Lactobacillus strains and increased the possibility of the presence of regulatory molecules in the bacterial EVs.

### 3.4. MSCl-Modulated moDC Differentiation Is Influenced by Lactobacillus-Derived Metabolites

As previously demonstrated, MSCl cells are able to modulate the differentiation, maturation, and functions of moDCs [23]. However, the priming of MSCs can fundamentally influence their immunomodulatory actions. Hence, we investigated whether *L. casei* CFS can modify the effect of MSCl cells on monocyte differentiation. Therefore, MSCl cells were exposed to EV-containing and UC *L. casei* CFSs for 24 h, and then the CFS-containing media were washed out and replaced with fresh media. The MSCl cells were activated with poly (I:C)-LyoVec complex for 48 h. The viral-ligand-containing medium was removed, and the cells were rested for an additional 48 h in fresh medium. After the incubation period, the MSCl-cell-conditioned media (MSCl-CM) were collected and added to freshly isolated human monocytes for 3 days (Figure 1D). 

**Figure 5:**
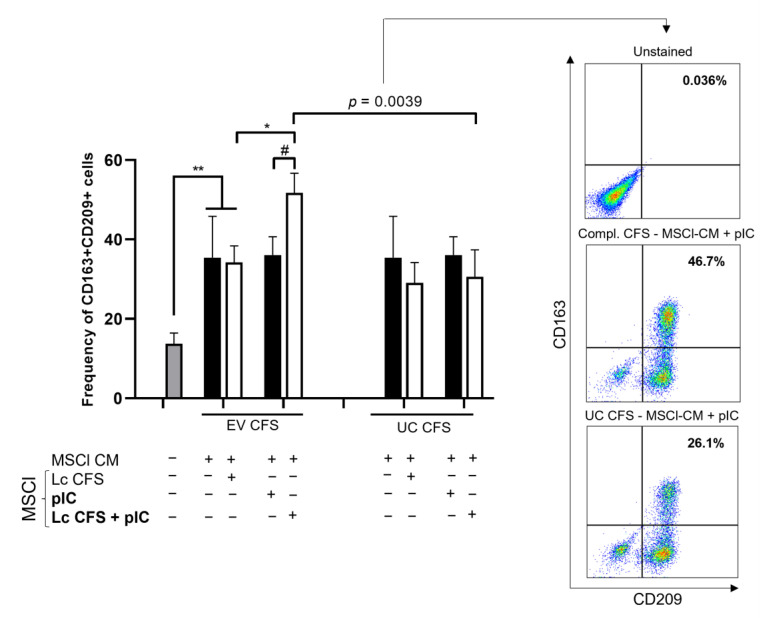
Bacterial vesicles can indirectly modulate the size of CD163^+^CD209^+^ moDC populations. A conditioned medium (CM) of EV and UC CFS-pretreated and poly (I:C)-activated MSCl cells was used at a ratio of 1:3 to culture monocyte-derived dendritic cells for 3 days, and the expression of CD163 and CD209 cell surface molecules was measured via flow cytometry. The values of the ratio of cells positive for the measured surface molecules were calculated based on 3 independent experiments and are presented as means ± SD. Two-way ANOVA with Tukey’s multiple comparison test was used for statistical analysis. Significance was defined as * *p* < 0.05 and ** *p* < 0.01 for the poly (I:C)-treated and untreated cells and # *p* < 0.05 for the comparison of the CFS-treated and untreated cells. A significant difference, defined as *p* = 0.0039, was established between CM derived from the EV-containing and UC CFS-treated MSCl cells upon poly (I:C) stimulation in the induction of CD163^+^CD209^+^ moDCs.

First, we monitored the changes in the expression of moDC differentiation markers, including CD209/DC-SIGN, CD163, CD14, CD86, and HLA-DQ, via flow cytometry (Figure 5 and Figure S2). We found that the presence of MSCl-CM increased the frequency of CD163^+^CD209^+^ moDCs. This MSCl-CM-induced enhancement was not altered by separate treatment of MSCl cells with *L. casei* CFS or poly (I: C). However, CM derived from the EV-containing *L. casei*-CFS-preconditioned and poly (I:C)-treated MSCl cells significantly enhanced the ratio of double-positive moDCs in comparison with CM from the EV-containing *L. casei*-CFS- or poly (I:C)-single-treated MSCl cells (Figure 5). Importantly, the depletion of EVs from *L. casei* CFS caused a significant reduction in its ability to cooperate with poly (I:C) activation in order to indirectly modify moDC differentiation. 

The expression of CD14 is constantly decreased on the surfaces of moDCs during differentiation [30], and this process was inhibited in the presence of MSCl-CM and not influenced by either Lactobacillus CFS or the viral stimulus (Figure S2A). Moreover, CD209 expression was not changed on the surfaces of moDCs in the presence of MSCl-CM, *L. casei* CFS, or the viral stimulus (Figure S2B). In line with this, none of the preconditions of the MSCl cells influenced the expression of co-stimulatory CD86 (Figure S2C) and antigen-presenting HLA-DQ molecules in the moDCs (Figure S2D).

Based on these results, the expression of CD163 is sensitive to the indirect effects of *L. casei*-CFS- and viral-stimulus-exposed MSCl cells, but this is not the case for the other moDC differentiation markers. Furthermore, the depletion of bacterial EVs works against the differentiation of CD163^+^ moDCs.

### 3.5. Cytokine/Chemokine Production by moDCs Is Indirectly Affected by MSCl Cells Exposed to Various Microenvironmental Signals

To study the MSCl-instructed moDC responses, we measured the concentrations of the cytokines—TNFα, IL-1β, IL-6, IFN-β, IL-10, IL-12, and TGFβ—and chemokines—IL-8 and CXCL10— secreted by moDCs treated with the CM of MSCl cells for 3 days. 

**Figure 6:**
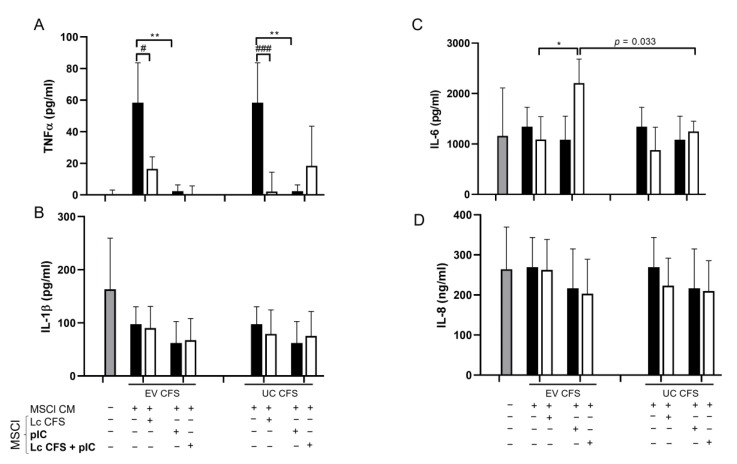
The cytokine and chemokine secretion of moDCs is indirectly modified by Lactobacillus CFS. MoDCs were cultured for 3 days in the presence of a conditioned medium (CM) derived from MSCl cells pretreated with ultracentrifuged (UC) and EV-containing supernatants from a Lactobacillus strain and activated with 1 µg/mL poly (I:C) synthetic viral ligand. The concentrations of TNFα (**A**), IL-1β (**B**), IL-6 (**C**), and IL-8 (**D**) in moDCs were determined via ELISA. Results from 3 independent measurements are presented as means ± SD. Two-way ANOVA with Tukey’s multiple comparison test was used for statistical analysis. Significance was defined as * *p* < 0.05 and ** *p* < 0.01 for the poly (I:C)-treated and untreated cells and # *p* < 0.05 and ### *p* < 0.001 for the CFS-treated and untreated cells. A significant difference (*p* = 0.033) was determined between CM derived from the EV-containing and UC CFS-treated MSCl cells upon poly (I:C) stimulation in the induction of IL-6 secretion by moDCs.

We found that the moDCs secreted more pro-inflammatory TNF-α when the cells were differentiated in the presence of CM obtained from non-treated MSCl cells. Both *L. casei* CFSs and the poly (I:C) treatment of MSCl cells indirectly induced significantly less TNFα secretion by the moDCs (Figure 6A). In addition to this, the moDCs independently produced IL-1β at the highest concentration. The addition of MSCl-CM, regardless of the type of MSCl treatment, caused slightly decreased IL-1β release by the moDCs (Figure 6B). Furthermore, the CM of MSCl cells treated with EV-containing *L. casei* CFS together with poly (I:C) promoted IL-6 production by the moDCs, an effect which was significantly reduced by EV depletion of the bacterial CFS (Figure 6C). The production of the IL-8 chemokine was not affected by the administration of non-treated or *L. casei*-CFS-treated MSCl-CM, while the poly (I:C) activation of MSCl cells induced slightly decreased IL-8 secretion by the moDCs (Figure 6D). In good agreement with the previous results of our research group [23], MSCl-CM induced higher IL-10 production by the moDCs. The EV-containing and UC *L. casei*-CFS-treated and poly (I:C)-stimulated MSCl cells indirectly instructed the moDCs to secrete more IL-10 than *L. casei* CFS alone (Figure S3A). We could not detect IL-12 or TGFβ production under any of the conditions tested (Figure S3B). 

**Figure 7:**
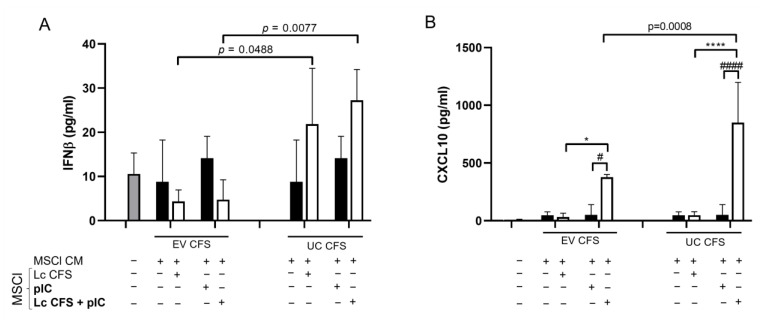
IFNβ and CXCL10 secretion is increased in MSCl-CM-exposed moDCs after the removal of vesicles from bacterial CFS. MSCl-cells were incubated in the presence of EV-containing and UC supernatants of *L. casei* for 24 h, activated with 1 µg/mL poly (I:C) for 48 h, and rested for another 48 h. Monocyte-derived dendritic cells were differentiated from monocytes in the presence of the collected conditioned medium (CM) derived from pretreated and activated MSCl cells for 3 days. The indirect effects of lactic acid bacteria-derived components on the secretion of IFNβ (**A**) and CXCL10 (**B**) by moDCs were measured via ELISA. Results from 3 independent measurements are presented as means ± SD. Two-way ANOVA with Tukey’s multiple comparison test was used for statistical analysis. Significance was defined as * *p* < 0.05 and **** *p* < 0.0001 in the comparison of poly (I:C)-treated and untreated cells and # *p* < 0.05 and #### *p* < 0.0001 in the comparison between CFS-treated and untreated cells. Significant differences between the EV and UC CFSs in different conditions are indicated by the calculated *p*-value.

Next, we aimed to analyze antiviral cytokine and chemokine secretion by moDCs differentiated in the presence of CM derived from MSCl cells exposed to *L. casei* CFS and the poly (I:C) stimulus. Interestingly, we found that CM derived from MSCl cells incubated with UC *L. casei* CFS—either alone or in co-treatment with poly (I:C)—induced significantly increased IFNβ secretion by the moDCs as compared to its EV-containing CFS counterpart (Figure 7A). Moreover, a significantly elevated CXCL10 level was observed in the moDCs differentiated in the presence of MSCl-CM when the MSCl cells were exposed to the two types of *L. casei* CFSs, as compared to the viral stimulus alone (Figure 7B). In agreement with the results obtained with MSCl cells (Figure 4C), the depletion of bacterial EVs caused significantly higher CXCL10 release by the moDCs (Figure 7B). 

A major function of DCs, as proficient antigen-presenting cells, is to prime and coordinate adaptive immune responses. IFNγ-producing T cells, i.e., CD4^+^ Th1 and CD8^+^ cytotoxic T lymphocytes, are crucial in viral infections. Therefore, the frequency of IFNγ-producing CD4^+^ and CD8^+^ T cells was analyzed using flow cytometry. Allogenic PBLs were treated with the supernatants collected from the moDCs differentiated in the presence of MSCl-CMs. We could not find differences between the numbers of CD4^+^ and CD8^+^ IFNγ-producing T cells in different conditions (Figure S4). The absence of IL-12 (Figure S3B) suggested the involvement of other essential soluble factors in the induction of Th1 and CD8^+^ T cell activation.

Taken together, our results showed that *L. casei* CFS induced a pronounced antiviral response from the MSCl cells without inducing inflammation. The removal of bacterial EVs modifies the antiviral and moDC-differentiating capacities of MSCl cells. 

## 4. Discussion

The complex interaction between immune and non-immune cells fundamentally influences the direction of immune responses to pathogens such as viruses. MSCs are resident non-immune cells found in various tissues and act as robust immunomodulatory cell types. These direct and indirect cell–cell interactions are further complicated by the presence of microbiota and its mediators on mucosal surfaces and skin.

Many Lactobacillus species have beneficial effects on pathogenic infections. *Lactobacillus acidophilus* acts on poly (I:C)-activated dendritic cells and upregulates defense genes against viruses [31]. In another study, three types of *L. mucosae* (1025, M104R01L3, and DCC1HL5) were investigated. They exerted different kinds of antiviral effects against the respiratory syncytial virus. *L. mucosae* M104R013L3 and enhanced the IFNβ and TNFα levels, whereas the DCC1HL5 treatment increased IL-1β and IL-10 production. All serotypes altered the composition of the mouse microbiota, but they had distinct effects on short-chain fatty acid levels in the gut. *L. mucosae* 1025 increased the acetate, butyrate, and propionate levels, whereas the M104R01L3 serotype only promoted acetate production [32]. Therefore, it can be concluded that Lactobacillus strains may have antiviral effects, but they act in a strain- and serotype-specific manner. Indeed, in our experiments, we could not observe any alterations in the mRNA expression of TLR3, RIG-I, and MDA5 receptors and type I IFNs in MSCl cells treated with *L. reuteri* CFS and activated with poly (I:C), as compared to poly (I:C)-activated control cells. Since *L. casei* and its EVs are widely used in research [33,34,35,36] and have anti-inflammatory effects in colitis [37,38], we aimed to investigate the impact of the cell-free supernatant of *L. casei* BL23 on the antiviral and immunomodulatory capacities of MSCl cells. 

It is well-known that Lactobacilli and their released factors, including metabolites such as lactate and acetate, antimicrobial molecules, fragments of the peptidoglycan, enzymes, and extracellular vesicles, exert remarkable, decisively anti-inflammatory, and antimicrobial effects on the host. However, immune cells such as dendritic cells can also be activated by the soluble Lactobacilli factors and produce pro-inflammatory mediators. In an in vitro study, *L. reuteri* supernatant induced the upregulation of CD80, CD86, and CCR7 and the secretion of TNFα, IL-1β, IL-6, IL-12, and IL-8 by bone-marrow (BM)-derived DCs [9]. In good agreement with this finding, in our experimental system, 5-day human moDCs expressed CD83 and CD86 at a higher level and produced TNFα, IL-1β, IL-6, and IL-8 upon *L. casei* CFS treatment. In contrast to this, the treatment of MSCl cells with *L. casei* CFS did not cause the secretion of TNFα and IL-1β but did induce the secretion of IL-6 and IL-8. The latter are readily produced by many types of MSCs, such as BM- [39], umbilical-cord- [40], and adipose-tissue-derived [41] MSCs in steady-state conditions. In a study by Pricola et al., it was demonstrated that IL-6 serves as an autocrine maintenance factor for BM-MSCs [42]. It was previously demonstrated that MSC-derived IL-6 has an immunosuppressive effect on, and contributes to the prostaglandin E2 production of, MSCs, inhibiting local inflammation in experimental arthritis [43]. In addition to its neutrophil chemoattractant activity, MSC-derived IL-8 has regulatory features in certain diseases, such as colorectal cancer, and promotes angiogenesis and proliferation [44]. 

Uncontrolled, systemic, pro-inflammatory cytokine secretion, i.e., cytokine storms in viral infections such as COVID-19 or severe influenza, can have life-threatening consequences. Therefore, it is crucial to balance the pro- and anti-inflammatory cells and factors. Because MSCl cells did not produce pro-inflammatory TNFα and IL-1β upon *L. casei* CFS treatment in our experimental system, we decided to use the MSC cell line in our further viral-infection-mimicking experiments. It has already been revealed that MSCs express a wide variety of PRRs, including viral nucleic-acid-sensing RIG-like and Toll-like receptors [45]. As it has also been demonstrated, the presence of PRR ligands can induce the increased expression of PRRs [26]. Confirming these results, in response to poly (I:C), MSCl cells expressed *TLR3*, *RIGI*, and *IFIH1/MDA5*, which were further increased by *L. casei* CFS exposure, indicating that *L. casei* secretes mediators enhancing the antiviral response of non-immune cells. This finding is in a good agreement with a novel study investigating the antimicrobial effects of *L. plantarum* CFS. The authors found that plantaricin 3 and 5 have antiviral effects against lentiviruses [46]. Since plantaricins are secreted exclusively by *L. plantarum*, other mediators from the *L. casei* secretome are responsible for the observed phenomena. Because *L. casei* is a facultative heterofermentative strain, glucose metabolism by the bacterium can lead to multiple end products, such as acetate, ethanol, and CO_2_, in addition to lactate [47].

It has recently been demonstrated that microbiota-derived short-chain fatty acid acetate can enhance the efficacy of antiviral immune responses against influenza A virus via both the RIG-I/MAVS-TBK1-IRF3 and GPR43/NLRP3-IRF3 pathways, promoting type I IFN production [48]. Similarly, lactate from lactic acid bacteria has also been shown to effectively influence antiviral responses in the vaginal mucosa with direct antiviral activity [49]. However, the roles of acetate and lactate in MSC-driven antiviral immunity have not yet been elucidated.

In addition to metabolic end products, other mediators can be identified in probiotic bacteria’s secretome, such as cell wall constituents including exopolysaccharides, lipoteichoic acid, peptidoglycan fragments, and enzymes. These molecules can also be found in and on the bacteria’s extracellular vesicles together with nucleic acids, antimicrobial molecules, and many other kinds of bioactive mediators [50]. The antimicrobial functions of the Lactobacillus EVs are often investigated in the context of bacterial infections; the antiviral mechanisms exerted by Lactobacillus EVs have barely been explored. In an ex vivo study using human tonsillar and cervico-vaginal tissues, it was reported that Lactobacillus EVs directly inhibited the attachment of HIV-1 to target cells [28]. In our experimental system, *L. casei* EV-containing CFS induced the enhanced production of IFNβ and CXCL10 by MSCl cells upon poly (I:C) stimulation. Surprisingly, the removal of EVs via ultracentrifugation further increased the amounts of these proteins, which may be explained by the fact that free metabolites such as acetate and lactate remained in the supernatant and the presence of regulatory factors in the bacterial EVs. The determination of the active metabolites in *L. casei* CFSs needs further investigations. 

MSC-derived soluble mediators are known to influence the differentiation, maturation, phenotypes, and functions of DCs. It has been reported that human BM-MSC-derived EVs reduced the expression of CD83 and CD80 by moDCs; however, CD86 and HLA-DR expression was unchanged [51]. In good agreement with this finding, in our experimental system, the expression of CD86 and HLA-DQ, along with CD14 and CD209, by the moDCs was not sensitive to the presence of MSCl-derived conditioned media. In contrast to this, we detected markedly increased CD163 expression in the moDCs by MSCl-CM, which was further intensified by CM derived from the *L. casei*-CFS- and poly (I:C)-treated MSCl cells. It has been demonstrated that CD14^+^CD163^+^CD141^+^ circulating DCs secrete IL-10 and IL-6 in a steady state, in addition to IL-12 and TNFα upon LPS stimulation [52]. In another study, CD163^+^ blood DCs were sensitive to TLR stimulations including poly (I:C); they produced TNFα, IL-1β, IL-12, IL-6, and IL-10 and induced the proliferation of CD8^+^CD103^+^ tissue-resident memory T cells [53], which may have antiviral activity [54]. The moDCs used in our in vitro model share similarities with the above-mentioned DCs. They produced TNF-α, IL-1β, IL-6, IL-8, and IL-10 but did not release IL-12 and TGFβ in the presence of MSCl-CM. Additionally, DCs could indirectly induce the activation of CD4^+^ Th1 and CD8^+^ T cells, though the different conditions did not influence this. The treatment of MSCl cells with both *L. casei* CFSs and poly (I:C) affected the composition of the MSCl-derived CM, which induced a drop in the moDCs’ TNF-α secretion and an elevation in IL-6 and IL-10 release, suggesting that the priming of MSCs with different kinds of factors can alter their immunomodulatory potential.

Despite the fact that the moDCs were not treated with a viral ligand directly, they could produce IFNβ and CXCL10 when they were differentiated in the presence of CM from *L. casei*-CFS- and poly (I:C)-treated MSCl cells. It has been demonstrated that MSC-derived exosomes can transport many kinds of molecules, such as micro-RNAs, small molecules such as doxorubicin and paclitaxel, and various proteins including cytokines and growth factors [55]. It can be postulated that in our system, the MSCl cells engulfed poly (I:C) and were then packed into exosomes and released into the environment, and thus, they could activate the moDCs’ antiviral response.

The MSCl cells used in our experimental system do not completely reflect the actions of primary MSCs. Since the functions of MSCs are greatly influenced by the tissue microenvironment, distinct tissue-derived primary MSCs may act differently in viral infections, as different MSCs have different functional properties [56]. Furthermore, poly (I:C), a synthetic viral ligand analogue, was applied to model a general viral infection. The use of different viruses may further clarify the antiviral properties of CFS-treated MSCl cells.

Taken together, our results showed that *L. casei* CFS enhanced the antiviral response of MSCl cells, in which Lactobacillus-derived EVs had a significant role. These MSCl cells indirectly induced the differentiation of anti-inflammatory moDCs, which retained their antiviral properties. Our findings may contribute to a deeper understanding of this topic in the field of licensed MSCs and be useful in regard to inflammatory disorders and viral infections.

## Data Availability

Not applicable.

## Supplementary Materials

The following supporting information can be downloaded at: https://www.mdpi.com/article/10.3390/biomedicines11061521/s1, Figure S1. Cell surface marker expression of moDCs is affected by *L. casei* CFS but MSCl marker expression is not. Figure S2: Expression of differentiation markers and cell surface markers involved in antigen presentation does not change in moDCs differentiated in the presence of MSCl-CM. Figure S3: MSCl-CM induces IL-10 secretion by moDCs but MSC-CM-exposed moDCs cannot produce IL-12 or TGF-β. Figure S4: MSCl-CM-exposed moDCs indirectly induce T cell activation but different conditions do not affect it.
