# Supplementary material for: Cell-Free Supernatant Derived from a Lactobacillus casei BL23 Culture Modifies the Antiviral and Immunomodulatory Capacity of Mesenchymal Stromal Cells
Source: Biomedicines. 2023 May 24;11(6):1521. doi: 10.3390/biomedicines11061521 (PMC10295444; doi:10.3390/biomedicines11061521)
Supplement: Supplementary file 1 [file biomedicines-11-01521-s001.zip › biomedicines-2350086-supplementary.pdf]

Supplementary Materials

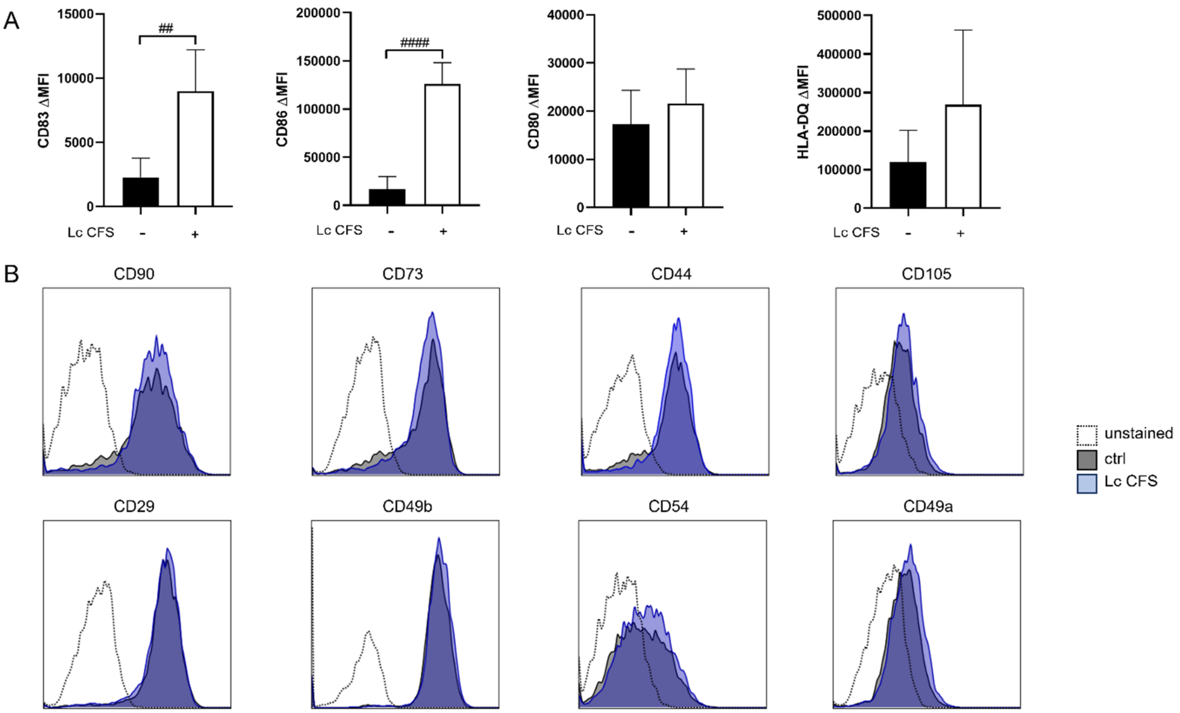

Figure S1. Cell surface marker expression of moDCs is affected by *L. casei* CFS but MSCl marker expression is not. MSCl cells and moDCs were exposed to *L. casei* CFS for 24 h and the expression of activation moDC markers (CD83, CD86, CD80 and HLA-DQ) (**A**) and MSCl markers (CD90, CD73, CD44, CD105, CD29, CD49b, CD54 and CD49a) (**B**) were checked by flow cytometry. The histograms show a representative result. Our results are from 5 independent measurements ± SD. Student's paired T-test was used for statistical analysis. Significance was defined as ## $p < 0.01$ and #### $p < 0.0001$ compared to CFS-untreated control cells.

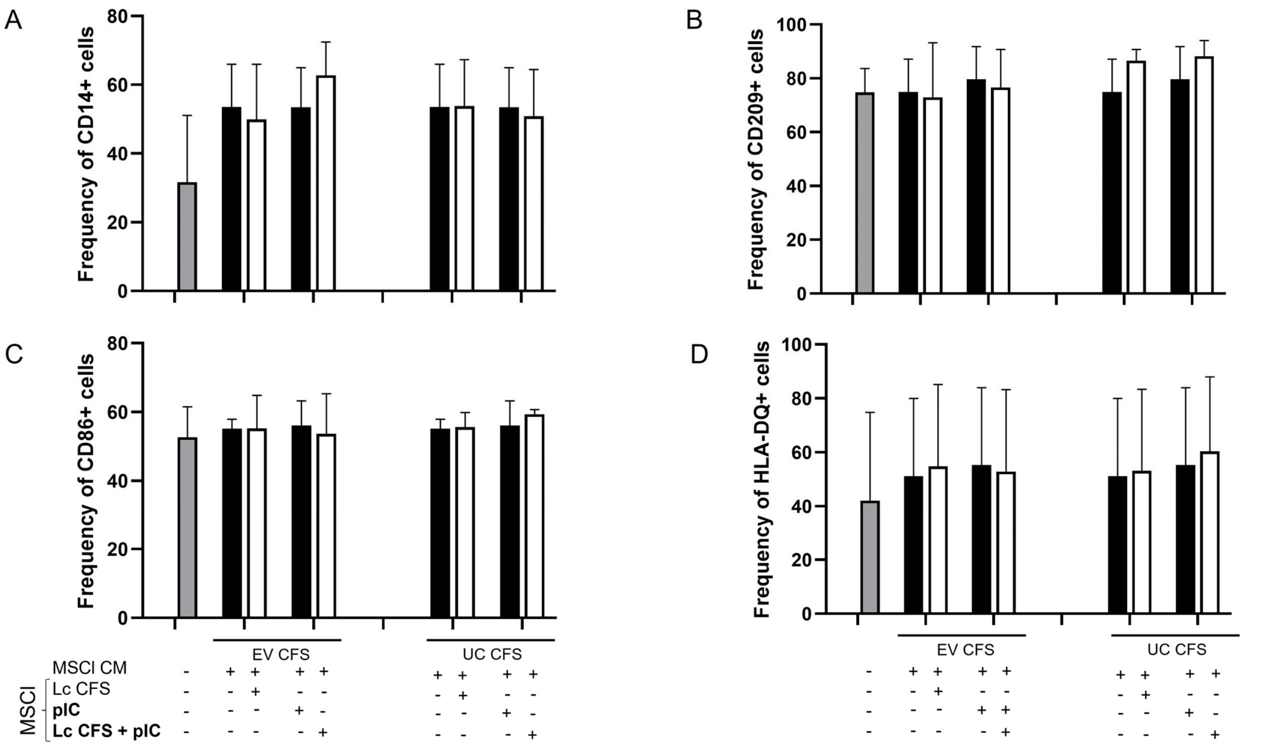

Figure S2. Expression of differentiation markers and cell surface markers involved in antigen presentation doesn't change by moDCs differentiated in the presence of MSCl-CM. Human moDCs were differentiated from CD14⁺ monocytes in the presence of GM-CSF, IL-4, and CM from MSCl cells exposed to EV-containing and UC CFS of *L. casei* and activated with 1 µg/ml poly (I:C). Changes in the expression of CD14 (**A**), DC-SIGN/CD209 (**B**), CD86 (**C**) and HLA-DQ (**D**) markers of moDCs were checked by flow cytometry. Mean values of the ratio of positive cells were calculated from 3 independent measurements ± SD. Two-way ANOVA with Tukey's multiple comparison test was used for statistical analysis.

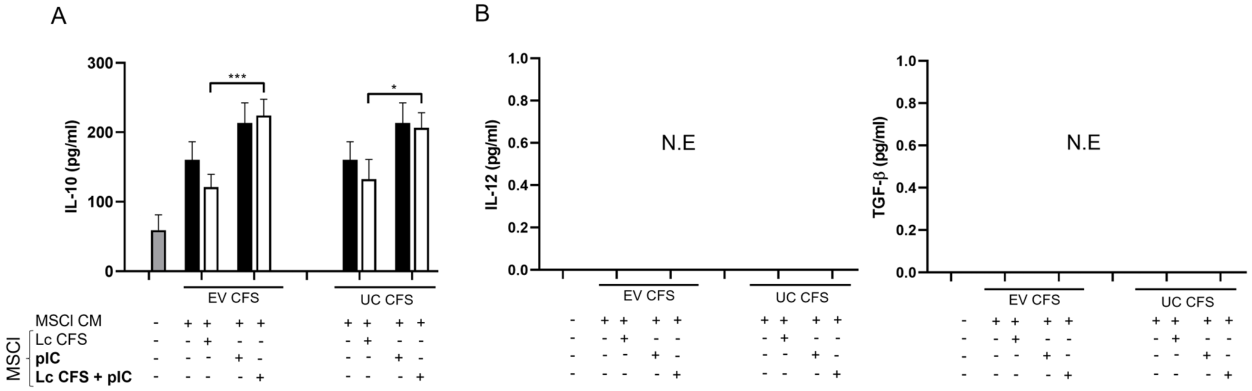

Figure S3. MSCl-CM induce IL-10 secretion by moDCs but MSC-CM-exposed moDCs cannot produce IL-12 and TGF-β. MSCl cells were pretreated with EV-containing and UC Lactobacillus CFSs for 24 h, then washed and activated with synthetic viral ligands for 48 h and cultured for another 48 h. The obtained MSCl-CM was used in the moDC differentiation for 3 days. IL-10 (**A**), IL-12 and TGF-β (**B**) production by moDCs was determined by ELISA. Our results are from 3 independent measurements ± SD. Two-way ANOVA with Tukey's multiple comparison test was used for statistical analysis. Significance was defined as * $p < 0.05$ and *** $p < 0.001$ between poly (I:C)-treated and untreated cells.

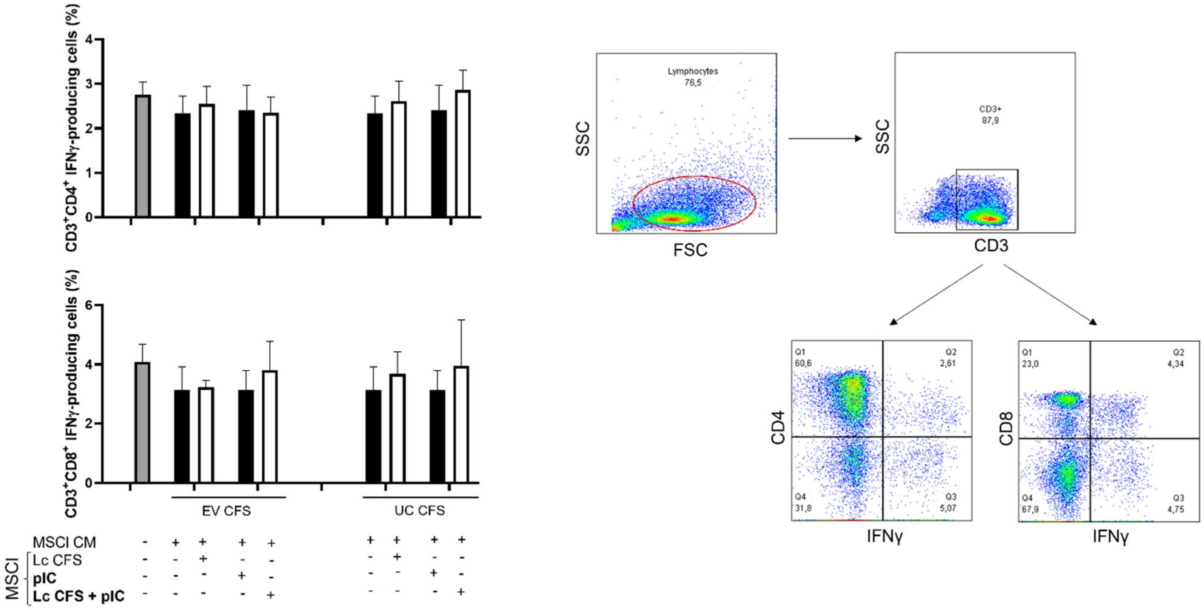

Figure S4. MSCl-CM-exposed moDCs indirectly induce T-cell activation but the different conditions do not affect it. MSC-like cells were pretreated with EV-containing and UC CFSs from *L. casei* for 24 h, then washed and activated with poly (I:C) synthetic viral ligand in complex with LyoVec transfection reagent for 48 h. The cells were washed and cultured another 48 h, and the obtained MSCl-CM were used to culture moDCs for 3 days. Supernatants of moDCs were used to treat allogenic PBLs for 72 h and activated with Ionomycin and PMA for 24 h. Bar plots show the frequency of IFNγ-producing $CD3^{+}CD4^{+}$ and $CD3^{+}CD8^{+}$ T cells, respectively. Our results are from 3 independent measurements ± SD. Dot plots on the right show the gating strategy used in the analysis of the flow cytometric data. Two-way ANOVA with Tukey's multiple comparison test was used for statistical analysis.
